# An Integrated Analysis of Transcriptomic and Metabolomic Effects Reveals Insights into Stress Responses in Largemouth Bass (*Micropterus salmoides*) Under MS-222 (Tricaine Methanesulfonate) Exposure

**DOI:** 10.3390/metabo15060349

**Published:** 2025-05-23

**Authors:** Ping Gao, Rimeng Chen, Deyun Ma, Shanshan Lin, Haodong Yu, Xuezhen Zhang

**Affiliations:** 1 College of Fisheries, Huazhong Agricultural University, Wuhan 430070, China; gaoyang2008694@126.com (P.G.); haodongyu@webmail.hzau.edu.cn (H.Y.); 2 Zhanjiang Institute for Food and Drug Control, Zhanjiang 524300, China; zjsys2014@126.com (R.C.); 18378915804@163.com (S.L.); 3 School of Food and Pharmaceutical Engineering, Zhaoqing University, Zhaoqing 526061, China; mady@zqu.edu.cn; 4 Engineering Research Center of Green Development for Conventional Aquatic Biological Industry in the Yangtze River Economic Belt, Ministry of Education, Wuhan 430070, China; 5 Hubei Provincial Engineering Laboratory for Pond Aquaculture, Wuhan 430070, China; 6 Hubei Hongshan Laboratory, Wuhan 430070, China

**Keywords:** MS-222, largemouth bass, transcriptomic, metabolomic

## Abstract

Background/Objectives: MS-222 is a commonly used anesthetic for fish. Research on the anesthetic mechanism of MS-222 is scarce, especially in largemouth bass. Therefore, this study investigated the tissue-specific transcriptomic and metabolomic effects of MS-222 anesthesia on largemouth bass (*Micropterus salmoides*). Methods: Experimental groups exposed to 40 mg/L MS-222 for 12 h were compared with untreated controls, and then transcriptomic and metabolomic analyses were performed on gill and liver samples. Results: Gill tissues exhibited 3252 differentially expressed genes (DEGs; 2309 upregulated and 943 downregulated) enriched in cardiac muscle contraction, cytoskeletal regulation, glycolysis, and toll-like receptor pathways for anesthetic adaptation. In contrast, liver tissues showed fewer DEGs (1140; 654 upregulated and 486 downregulated) primarily linked to metabolic network reorganization such as endoplasmic reticulum protein processing, PPAR signaling, and ribosome biogenesis. Metabolomic profiling demonstrated inverse patterns, with 173 differential metabolites in gills versus 297 in liver samples. Methyl nicotinate and N-acetyl-L-phenylalanine were the most significantly upregulated in the gill and liver samples. Metabolic pathway enrichment analysis revealed that MS-222-induced differential metabolites in the gill and liver of largemouth bass were predominantly associated with pathways involved in amino acid, fatty acid, phenylalanine, and nucleotide metabolism. Conclusions: These findings reveal that MS-222 anesthesia triggers organ-specific physiological adaptations through the differential regulation of metabolic and immune pathways, which provide multi-omics insights into the mechanistic basis of anesthetic responses in fish, highlighting distinct tissue strategies for managing chemical stress.

## 1. Introduction

The largemouth bass (*Micropterus salmoides*), prized for its rapid growth, superior flesh quality, and significant market value, has become a cornerstone species in China’s freshwater aquaculture industry. In 2023 alone, domestic production reached 888,030 tons [1]. However, the transportation process poses significant challenges to fish welfare, with stress-induced physiological disturbances adversely affecting survival rates, growth performance, and product quality [2]. To address these challenges in the transportation of live aquatic products, the strategic application of fish anesthetics has emerged as a critical solution. These agents effectively reduce metabolic demands, suppress oxygen consumption, and mitigate stress responses through the modulation of the hypothalamic–pituitary–adrenal (HPA) axis [3] while minimizing physical trauma during handling operations.

Currently, more than 30 different types of fish anesthetics are available, including 3-aminobenzoic acid ethyl ester methanesulfonate (MS-222), clove oil, and carbon [4]. MS-222, renowned for its effects and safety, is approved by the Food and Drug Administration (FDA) for use on food fish in the USA and European Union [5,6,7,8]. In recent decades, extensive research has documented MS-222’s anesthetic efficacy and physiological impacts across diverse teleost species, including red drum *Sciaenops ocellatus* [9], Atlantic salmon *Salmo salar* [10], pikeperch *Sander lucioperca* [11,12], rainbow trout *Oncorhynchus mykiss* [13], largemouth bass *Micropterus salmoides* [14], spotted *knifejaw Oplegnathus* punctatus [15], Chinese sea bass *Lateolabrax maculatus* [16,17], and yellow catfish *Pelteobagrus fulvidraco* [18]. However, research on the anesthetic mechanism of MS-222 is still insufficient.

With the development of sequencing technology, omics technology has been increasingly used in a broad range of applications for analyzing the molecular mechanisms of aquatic animals’ responses to external factors. While transcriptomic approaches have elucidated anesthetic mechanisms in crucian carp and Chinese sea bass [19,20,21], significant knowledge gaps persist regarding MS-222’s systemic effects in largemouth bass. Meanwhile, metabolomic changes have not been reported in largemouth bass exposed to MS-222. Notably, the pharmacokinetic profile of MS-222, characterized by rapid gill absorption, systemic distribution, and hepatic accumulation [22], necessitates a comprehensive evaluation of its molecular impacts on these target tissues. Therefore, in this study, we employed a combination of transcriptomic and metabolomic analyses to investigate gill and liver responses to MS-222 exposure. Using a multi-omics approach, we aimed to identify the key molecular pathways involved in the modulation and characterization of tissue-specific metabolic effects upon anesthetic exposure, which could advance our understanding of the underlying mechanisms of piscine anesthesia.

## 2. Materials and Methods

### 2.1. Fish Husbandry

The largemouth bass species were obtained from a fish farm in Zhanjiang city, Guangdong Province, PR China, and acclimated to cylindrical fiberglass tanks (diameter = 1.0 m) for 2 weeks. During the acclimation period, the fish were fed a commercial diet twice daily at 08:00 and 18:00 until satiation, with the photoperiod set to 16 h of light and 8 h of darkness. Water temperature, dissolved oxygen, pH, and ammonia nitrogen levels were regularly monitored. The water temperature ranged from 23.0 °C to 25.0 °C, the pH varied from 7.5 to 8.0, ammonia nitrogen was maintained below 0.1 mg/L, and dissolved oxygen remained above 5.0 mg/L.

### 2.2. Experimental Design and Sampling

Based on the results of our previous study, we selected an MS-222 solution (Shanghai Aladdin Biochemical Technology Co., Ltd., Shanghai, China) with a concentration of 40 mg/L, which induced the fish to undergo Stage III anesthesia (deep sedation) and has been determined to be suitable for anesthetic transport under experimental conditions [23]. Consequently, an additional 60 fish were randomly distributed into 6 tanks (100 L), with 10 individuals per tank. An MS-222 anesthesia group (40 mg/L) and a control group were established with 6 replicates (labeled as MA and CA, respectively). After 12 h of deep sedation, 3 fish were randomly selected from each tank and euthanized quickly by a blow to the head. Then, the fish were dissected, and the gill and liver samples were separated and stored in liquid nitrogen.

### 2.3. Transcriptomic Analysis

#### 2.3.1. RNA Extraction

Total RNA was extracted from gill and liver samples using TRIzol (Invitrogen, Carlsbad, CA, USA). Briefly, approximately 50–100 mg of tissue was homogenized in 1 mL of TRIzol, followed by phase separation with chloroform and RNA precipitation with isopropanol. The RNA pellet was washed with 75% ethanol, air-dried, and dissolved in RNase-free water. Then, the degradation and contamination of RNA were monitored on 1% agarose gels. RNA purity, concentration, and integrity were assessed using a NanoPhotometer spectrophotometer (Implen Inc., Westlake village, CA, USA), Qubit RNA Assay Kit in Qubit 2.0 Flurometer (Life Technologies, Carlsbad, CA, USA), and the Bioanalyzer 2100 system equipped with an RNA Nano 6000 Assay Kit (Agilent Technologies, Santa Clara, CA, USA), respectively.

#### 2.3.2. Library Preparation for Transcriptomic Sequencing

For sample preparation, l ug of RNA was used as input per sample. Sequencing libraries were generated using the NEB NextR Ultra TMRNA Library Prep Kit for llumina (NEB, Ipswich, MA, USA) following the manufacturer’s recommendations, and index codes were added to attribute sequences to each sample. The library quality was assessed on the Agilent Bioanalyzer 2100 system.

#### 2.3.3. Clustering and Sequencing

The index-coded samples were clustered on a cBot Cluster Generation System using TruSeq PE Cluster Kit v3-cBot-HS (Illumina, San Diego, CA, USA) according to the manufacturer’s instructions. Briefly, the pooled RNA libraries were denatured with NaOH, diluted to 8 pM with pre-chilled hybridization buffer, and combined with 1% (*v*/*v*) PhiX Control V3 Library as an internal control. The mixture was then loaded onto the flow cell and amplified using bridge PCR on the cBot system under the following conditions: 98 °C for 30 s (initial denaturation), followed by 10 cycles of 98 °C for 10 s (denaturation), 60 °C for 30 s (annealing), and 72 °C for 30 s (extension), with a final extension at 72 °C for 5 min. After clustering, the flow cell was transferred to the Illumina sequencing platform for paired-end sequencing, which included (1) initial primer hybridization and first base incorporation; (2) cyclic reversible termination with fluorescently labeled nucleotides; (3) imaging acquisition for base calling at each cycle; and (4) strand regeneration for the subsequent read. Quality control metrics, namely Q30 scores (>80%) and cluster density (optimal range: 170–220 K/mm^2^ for NovaSeq), were monitored throughout the process. The raw image data were processed in real time using Illumina’s RTA v1.18 (Real-Time Analysis) software for base calling and converted to the FASTQ format using bcl2fastq (v2.20) with default parameters, generating paired-end reads (Read1: 150 bp and Read2: 150 bp) with the corresponding index sequences.

#### 2.3.4. Data Processing

The raw data were trimmed and quality-controlled using fastp v0.23.2 with default parameters. The clean reads were mapped to the reference genome of *M. salmoides* (GCF_014851395.1_ASM1485139v1_genomic.fna, GCF_014851395.1_ASM1485139v1_genomic.gff) using the designated databases; namely, the NCBI non-redundant (NR) protein database, Gene Ontology (GO), Clusters of Orthologous Groups (COG), and Kyoto Encyclopedia of Genes and Genomes (KEGG). Then, the reads were aligned to the reference genome using HISAT2 v2.2.1, a highly efficient splice-aware aligner, to determine their genomic positions and annotate sample-specific sequence features. DESeq2 v1.40.2 was used to analyze the differential expression between the two groups, and the *p*-value was corrected using the Benjamini and Hochberg method. |log2foldchang| ≥ 1 and a false discovery rate (FDR) of ≤0.05 were considered significantly differentially expressed genes in the samples.

### 2.4. Metabolomic Analysis

#### 2.4.1. Sample Preparation and Extraction

The refrigerated samples stored at −80 °C were thawed on ice for 5~10 min and then homogenized using a grinder (30 HZ) for 20 s. A 400 μL solution (methanol–water = 7:3, *v*/*v*) containing internal standard (detailed information is shown in Table S1) was added to 20 mg of the ground sample, and the mixture was shaken at 1500 rpm for 5 min. After placing the samples on ice for 15 min, they were centrifuged at 12,000 rpm for 10 min (4 °C). Then, 300 μL aliquots of the supernatant were collected and placed in a refrigerator at −20 °C for 30 min, followed by centrifugation at 12,000 rpm for 3 min (4 °C). Then, 200 μL aliquots of the supernatant were transferred for LC-MS analysis.

#### 2.4.2. HPLC Conditions

All samples were eluted from the T3 column (Waters ACQUITY Premier HSS T3 Column 1.8 µm, 2.1 mm × 100 mm) using 0.1% formic acid in water as solvent A and 0.1% formic acid in acetonitrile as solvent B in the following gradient: 5 to 20% in 2 min, increased to 60% in the following 3 min and 99% in 1 min, held for 1.5 min, and then reduced to 5% mobile phase B within 0.1 min and held for 2.4 min. The analytical conditions were as follows: column temperature, 40 °C; flow rate, 0.4 mL/min; injection volume, 4 μL.

#### 2.4.3. MS Conditions (AB)

Data acquisition was performed using the information-dependent acquisition (IDA) mode in the Analyst TF 1.7.1 Software (Sciex, Concord, ON, Canada). The source parameters were set as follows: ion source gas 1 (GAS1), 50 psi; ion source gas 2 (GAS2), 50 psi; curtain gas (CUR), 25 psi; temperature (TEM), 550 °C; declustering potential (DP), +60 V/−60 V; and ion spray voltage floating (ISVF), +5000 V/−4000 V. The TOF MS scan parameters were set as follows: mass range, 50–1000 Da; accumulation time, 200 ms; and dynamic background subtract, on. The product ion scan parameters were set as follows: mass range, 25–1000 Da; accumulation time, 40 ms; collision energy, 30 or −30 V in positive or negative modes, respectively; collision energy spread, 15; resolution, UNIT; charge state, 1 to 1; intensity, 100 cps; exclude isotopes within 4 Da; mass tolerance, 50 ppm; maximum number of candidate ions to monitor per cycle, 18.

#### 2.4.4. Data Processing

The original data file generated using LC-MS was converted into the mzML format using ProteoWizard. Peak extraction, peak alignment, and retention time correction were, respectively, performed using the XCMS program. The “SVR” method was used to correct the peak area. Peaks with a detection rate lower than 50% in each group of samples were discarded. Then, metabolic identification data were obtained by searching the laboratory’s self-built database, as well as an integrated public database, an AI database, and metDNA. Differential metabolites were determined using VIP (VIP > 1) and *p*-value (*p* < 0.05, Student’s *t*-test).

## 3. Results

### 3.1. Transcriptomic Analysis

#### 3.1.1. Transcriptomic Sequence Assembly

The raw reads of each sample ranged from 46,078,140 to 54,862,588, the number of clean reads ranged from 43,466,010 to 53,770,872 after filtration, and the total number of bases was 6.49~7.6 G. The overall sequencing error was 0.03%. The number of bases with Qphred values above 20% and 30% accounted for 96% and 91% of the total number of bases, indicating relative reliability considering the base identification error. In addition, the total number of G and C bases accounted for 45.4% to 48.88% of the total number of bases (Table 1).

#### 3.1.2. Annotation and Functional Analyses

After clean data were obtained, the reference genomes (*M. salmoides*) were compared. The number of reads mapped to the reference genome, the unique reference genome, and multiple reference genomes are shown in Table 2. All samples exhibited high alignment rates, with >92% of the reads mapped to the reference genome and >83% uniquely aligned.

#### 3.1.3. Differentially Expressed Genes

**Figure 1:**
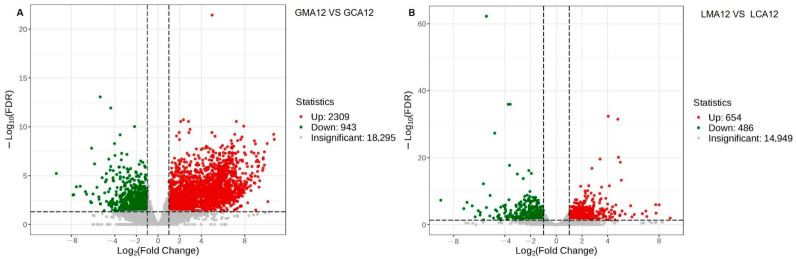
Volcano Plot of Differential genes Between Different Experimental Groups in Gill (**A**) and Liver (**B**) ^1^. ^1^ CA, Control group; MA, MS-222 treated group.

Transcriptional profiling at 12 h post-MS-222 anesthesia revealed stark tissue-specific responses. Gill tissues exhibited pronounced genomic alterations, with RNA-Seq analysis identifying 3252 differentially expressed genes (DEGs; 2309 upregulated and 943 downregulated), as shown in Figure 1A. In striking contrast, liver tissues demonstrated a less pronounced transcriptional response, displaying only 1140 DEGs (654 upregulated and 486 downregulated, Figure 1B).

**Figure 2:**
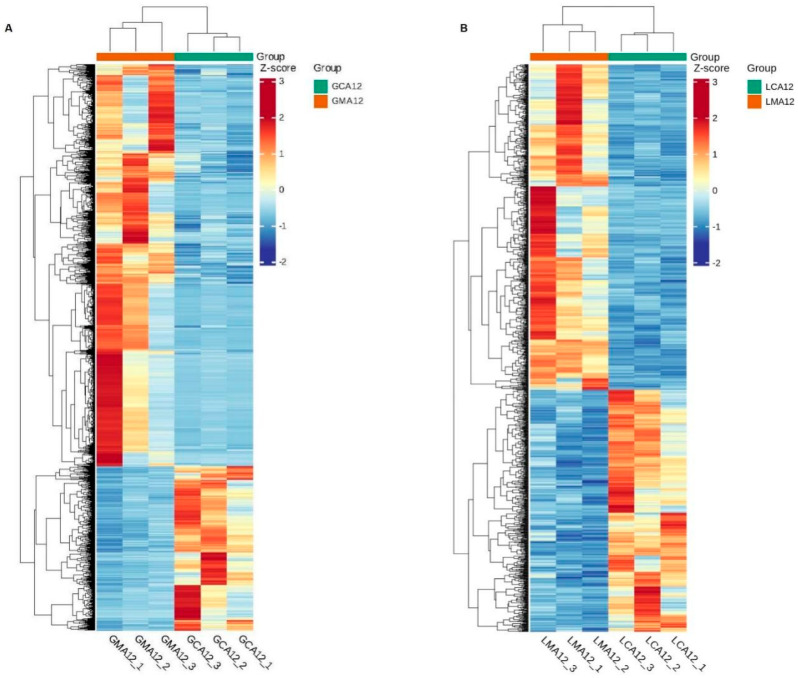
The clustering heatmap of differentially expressed genes in gill (**A**) and liver (**B**) tissues ^1^. ^1^ CA, Control group; MA, MS-222 treated group.

Heatmap visualization revealed pronounced segregation in transcriptional profiles, with clear separation between MS-222-treated and control groups across both gill (Figure 2A) and liver (Figure 2B) tissues, suggesting that MS-222 has a marked effect on the transcriptional levels of gill and liver tissues in largemouth bass.

**Figure 3:**
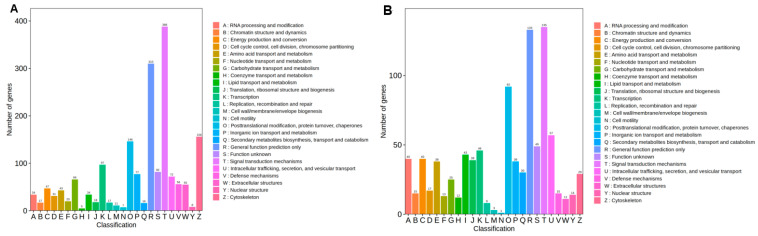
KOG annotation of differentially expressed genes in gill (**A**) and liver (**B**).

The functional annotation of DEGs against the KOG (euKaryotic Orthologous Groups) database revealed 23 defined functional categories in gill tissues, excluding the “general function prediction only” and “function unknown” classifications. The predominant categories included mechanisms associated with signal transduction, cytoskeleton, post-translational modification, protein folding and chaperones, transcription, and inorganic ion transport and metabolism (Figure 3A, Table S2). In liver tissues, the proportional distribution of functional categories mirrored those observed in gill tissues, with the notable exception of cytoskeleton-related processes. However, the absolute number of annotated genes in each category was substantially lower in liver than in gill tissues (Figure 3B, Table S3).

**Figure 4:**
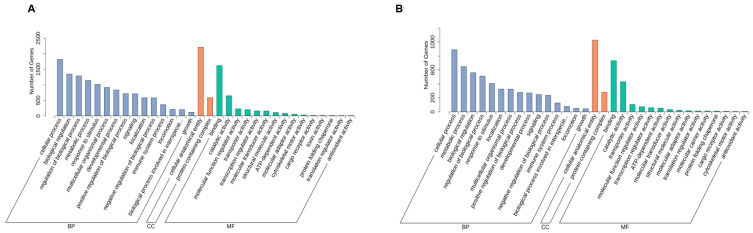
GO annotation and classification of all uniquely identified genes in gill (**A**) and liver (**B**) ^1^. ^1^ CA, Control group; MA, MS-222 treated group.

The GO enrichment analysis of differentially expressed genes (DEGs) in gill and liver tissues revealed distinct functional categorization across three major ontologies: biological process [BP], cellular component [CC], and molecular function [MF] (Tables S4 and S5). The top 15 enriched terms are illustrated in Figure 4. In gill tissues, the most prominently enriched BPs included cellular mechanisms, the regulation of biological processes, metabolic processes, and response to stimuli. Significant enrichment was observed for the protein-containing complex and the cellular anatomical entity in the CC category, whereas binding, catalytic activity, molecular function, regulator activity, and transporter activity were the largest MF subcategories (Figure 4A, Table S6). In liver tissues, the GO annotation classifications of the DEGs were similar to those of the gill tissues with lower gene numbers (Figure 4B, Table S7).

**Figure 5:**
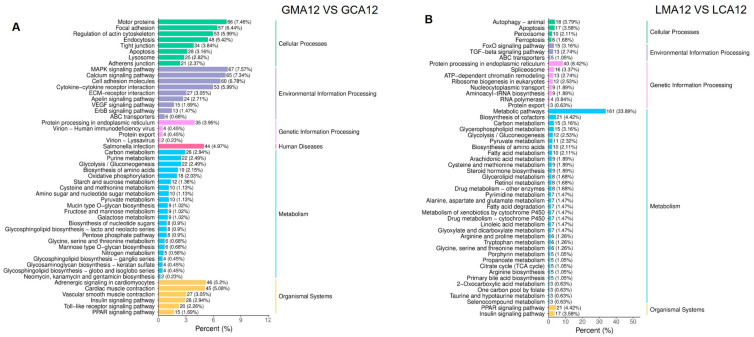
KEGG enrichment histogram of differentially expressed genes in gill (**A**) and liver (**B**) ^1^. ^1^ CA, Control group; MA, MS-222 treated group.

The 50 most significantly enriched pathways were selected to generate the KEGG enrichment column chart. Details of the number of DEGs annotated against the KEGG database and their ratio to the total number of DEGs are shown in Figure 5A,B. In the gill tissues, the dominant DEGs were annotated to pathways related to cellular processes (332 genes), environmental information processing (330 genes), metabolism (231 genes), and organismal systems (179 genes). In contrast, in liver tissues, the number of DEGs associated with metabolic pathways was up to 410, followed by genetic information processing (106), cellular processes (53 genes), organismal systems (38 genes), and environmental information processing (33 genes).

**Figure 6:**
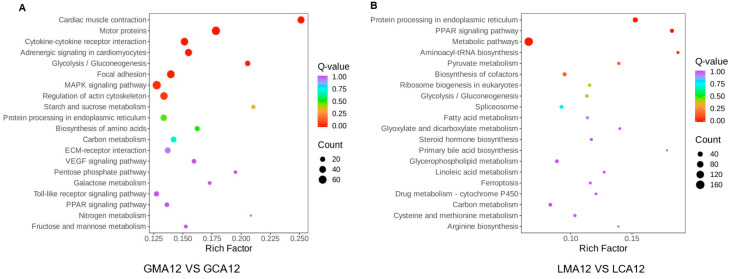
KEGG enrichment sites of differentially expressed genes in gill (**A**) and liver (**B**) ^1^. ^1^ CA, Control group; MA, MS-222 treated group.

The 20 most significantly enriched pathways were selected to generate the bubble map of KEGG enrichment analysis (Figure 6). The detailed information, including description, gene ratio, and the number of upregulated and downregulated genes, is shown in Tables S8 and S9. In gill samples, the DEGs exhibited significant enrichment in several key biological pathways, including cardiac muscle contraction, motor protein activity, adrenergic signaling in cardiomyocytes, glycolysis/gluconeogenesis, MAPK signaling, actin cytoskeleton regulation, cytokine–cytokine receptor interaction, and toll-like receptor signaling (Figure 6A). In liver tissues, the DEGs demonstrated significant enrichment in fundamental biological processes, particularly those associated with metabolic regulation and biosynthetic mechanisms. As illustrated in Figure 6B, the most prominently enriched pathways were associated with protein processing in the endoplasmic reticulum, PPAR signaling, metabolism, aminoacyl–tRNA biosynthesis, pyruvate metabolism, cofactor biosynthesis, ribosome biogenesis in eukaryotes, glycolysis/gluconeogenesis, spliceosome function, fatty acid metabolism, steroid hormone biosynthesis, primary bile acid biosynthesis, and linoleic acid metabolism, among others.

### 3.2. Metabolomic Analysis

#### 3.2.1. Gill

**Figure 7:**
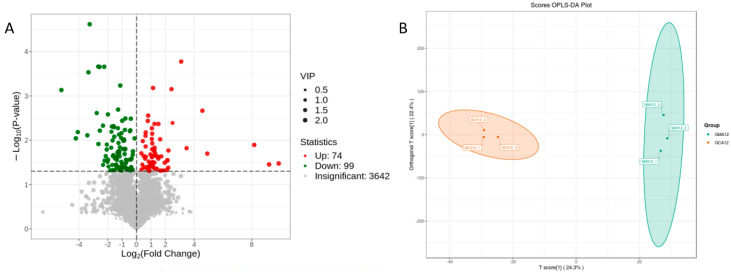

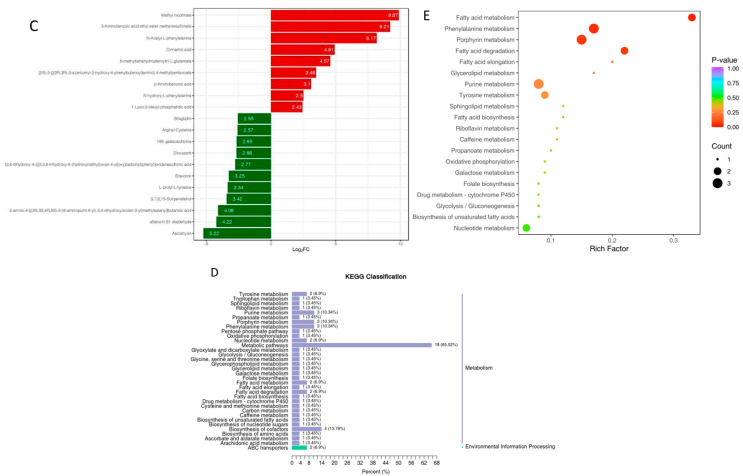
The metabolomic changes in the gill of the largemouth bass after 12 h of exposure to MS-222: (**A**) Volcano plot of differential metabolites; (**B**) PLS-DA results of metabolite abundance; (**C**) Log2FC of differential metabolites (top 20); (**D**) KEGG classification map of differential metabolites; (**E**) KEGG enrichment analysis of differential metabolites in the gill tissues of the different experimental groups ^1^. ^1^ CA, Control group; MA, MS-222 treated group.

A total of 3815 metabolites were identified in the gill samples. Compared to the control samples, 74 serum metabolites were significantly upregulated (*p* < 0.05), while 99 were significantly downregulated (*p* < 0.05) in the MA bass samples (Figure 7A). Details of the differential metabolites are shown in Table S10.

PLS-DA analysis revealed that the metabolites in the MS-222-treated group were separated from those in the control group, and the samples in the same treatment group were clustered together, indicating that MS-222 markedly changed the metabolites of the gill tissues in largemouth bass (Figure 7B).

We focused on the top 20 different metabolites of the MA and CA groups. Based on the quantitative metabolite information from these two groups, a bar chart was generated to illustrate the top 20 metabolites exhibiting the most significant fold changes (Figure 7C). Among them, methyl nicotinate, 3-aminobenzoic acid ethyl ester methanesulfonate, N-acetyl-L-phenylalanine, cinnamic acid, 5-methyltetrahydropteroyltri-L-glutamate, (2S)-2-[[(2R,3R)-3-azaniumyl-2-hydroxy-4-phenylbutanoyl]amino]-4-methylpentanoate, p-aminobenzoic acid, N-hydroxy-L-phenylalanine, and 1-Lyso-2-oleoyl-phosphatidic acid exhibited increased levels. Conversely, a decrease was observed in sitagliptin, arginyl–cysteine, 19E-geissoschizine, dioxacarb, [2,6-dihydroxy-4-({[2,3,5-trihydroxy-6-(hydroxymethyl)oxan-4-yl]oxy}carbonyl)phenyl]oxidanesulfonic acid, braxoron, L-prolyl-L-tyrosine, 3,7,8,15-scirpenetetrol, 2-amino-4-1(2S,3S,4R,5S)-5-(6-aminopurin-9-y1)-3,4-dihydroxyoxolan-2-yl]methylselanyl]butanoic acid, aflatoxin B1 dialdehyde, and ascomycin.

According to the annotation results, the metabolites exhibiting significant differences were categorized based on the pathway types outlined in the KEGG database and are illustrated in Figure 7D. The functional categorization of these differential metabolites reveals a prominent enrichment in metabolic pathways. KEGG (Kyoto Encyclopedia of Genes and Genomes) pathway enrichment analysis was performed on the top 20 pathways ranked by *p*-value based on the differential metabolites’ data, as presented in Figure 7E. Compared to the control (CA) samples, in the MA samples, serum differential metabolites exhibited the most significant enrichment in the metabolic pathways of fatty acids, phenylalanines, porphyrin, glycerolipids, purine, tyrosine, sphingolipid, riboflavin, caffeine, propanoate, galactose, and nucleotides, as well as the pathways associated with fatty acid degradation, elongation, and biosynthesis; oxidative phosphorylation; folate biosynthesis; drug metabolism through cytochrome P450 expression; glycolysis/gluconeogenesis; and the biosynthesis of unsaturated fatty acids.

#### 3.2.2. Liver

**Figure 8:**
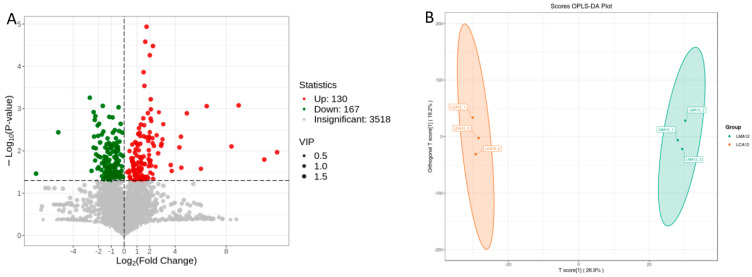

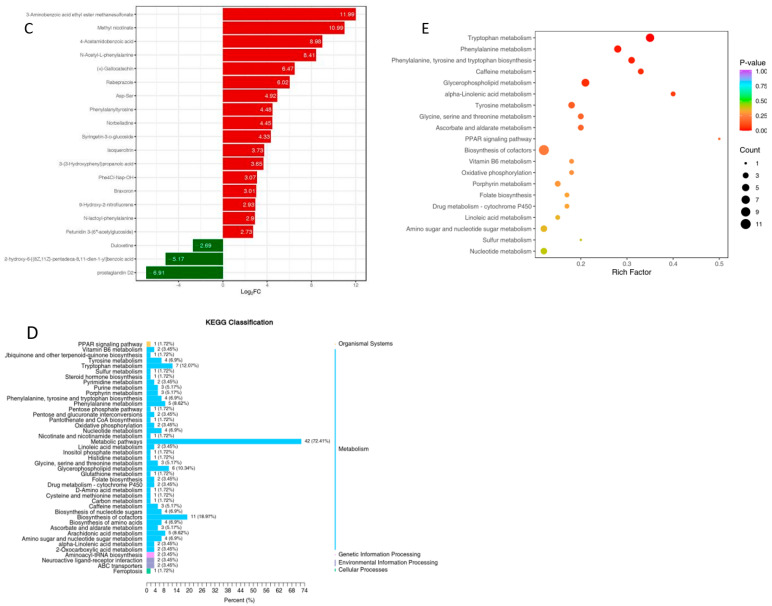
The metabolomic changes in the liver of the largemouth bass after 12 h of exposure to MS-222: (**A**) Volcano plot of differential metabolites; (**B**) PLS-DA results of metabolite abundance; (**C**) Log2FC of differential metabolites (top 20); (**D**) KEGG classification map of differential metabolites; (**E**) KEGG enrichment analysis of differential metabolites in the liver tissues of the different experimental groups ^1^. ^1^ CA, Control group; MA, MS-222 treated group.

Compared to the control samples, 130 serum metabolites were significantly upregulated (*p* < 0.05), and 167 were significantly downregulated (*p* < 0.05) in the MA bass samples (Figure 8A). Details of differential metabolites are illustrated in Table S11.

PLS-DA analysis indicated that metabolites in the MS-222-treated group were separated from those in the control group, and the samples in the same treatment group were clustered together (Figure 8B).

The top 20 metabolites exhibiting the most significant fold changes are presented in Figure 8C. Metabolites such as 3-Aminobenzoic acid ethyl ester methanesulfonate, Methyl nicotinate, 4-Acetamidobenzoic acid, N-Acetyl-L-phenylalanine, (+)-galocatechin, Rabeprazole, Asp-Ser, phenylalanyityrosine, norbelladine, Syringetin-3-o-glucoside, Isoquercitrin, 3-(3-Hydroxypheny) propanoic acid, Phe4CI-Nap-OH, Braxoron, 9-Hydroxy-2-nitrofluorene, N-lactoyl-phenylalanine, and petunidin 3-(6″-acetyiglucoside) were upregulated in the MA group, while those of Duloxetine, 2-hydroxy-6-[(8Z,11Z)-pentadeca-8,11-dien-1-yl]benzoic acid, and prostaglandin D2 were downregulated (Figure 8C).

The KEGG annotation results in the liver tissues are similar to those in the gill samples, indicating that most of the differential metabolites are involved in metabolism, as shown in Figure 8D. The KEGG signal pathway enrichment analysis of the different metabolites between the two groups revealed that the differential metabolites were mainly involved in the metabolism of tryptophan, phenylalanine, phenylalanine, caffeine, glycerophospholipids, alpha-linolenic acid, tyrosine, glycine, serine–threonine, ascorbate and aldarate, vitamin B6, porphyrin, linoleic acid, amino and nucleotide sugars, sulfur, and nucleotides, as well as tyrosine and tryptophan biosynthesis, PPAR signaling, cofactor biosynthesis, oxidative phosphorylation, folate biosynthesis, and drug metabolism through cytochrome P450 expression (Figure 8E).

## 4. Discussion

### 4.1. Transcriptomic Response Profiles

#### 4.1.1. Global Gene Expression Patterns

In aquaculture, organisms are frequently exposed to a myriad of stressors, including environmental changes, transportation-induced vibrations, manual operation, temperature variations, and alterations in stocking density. These stressors can trigger significant physiological and biochemical alterations in aquatic species, ultimately impacting their overall health and growth performance [24]. To mitigate the stress responses caused by manual operation, MS-222, a commonly utilized anesthetic in fisheries, is extensively employed for various purposes such as transportation, surgical procedures, spawning induction, and experimental studies involving aquatic organisms [25]. While MS-222 has demonstrated notable efficacy in alleviating stress responses in aquatic species, research elucidating its effects on the transcriptomic profiles of fish remains scarce. Nevertheless, it has been established that MS-222 exerts an influence on the transcriptome of aquatic organisms, as evidenced by its effects on physiological and biochemical parameters [25,26], as well as behavioral responses in fish [27,28,29].

In recent years, several studies have begun to focus on the effects of MS-222 on the transcriptome in fish. Cao et al. [19] and Le et al. [20] analyzed the transcriptome of blood samples when crucian carp species were exposed to two different anesthetics, MS-222 (100 mg/L) and eugenol (20 mg/L), respectively. A total of eight differentially expressed genes (DEGs) related to the immune response were co-expressed in the two anesthetic groups. GO and the KEGG analyses revealed that these genes were primarily associated with the enrichment of antigen processing and presentation pathways, including major histocompatibility complex I (MHCI), MHCIα, p-MHCIIα, P-MHCIIα, integrin-associated protein CD74, MHCII antigen, and MHCII antigenα. Moreover, LRRFIP2 was found to be associated with the immune response [19]. Le et al. identified 487 DEGs in the group treated with 100 mg/mL MS-222 (179 upregulated and 308 downregulated genes), including 155 DEGs related to stress responses, mainly to oxidative stress, heat shock proteins, and cold shock domain-containing proteins. The heat shock protein genes were the primary DEGs in response to stress. Investigating Chinese sea bass (*Lateolabrax maculatus*) [20], Dong et al. identified 4708 DEGs in the fish exposed to MS-222 (60 mg/mL), comprising 2513 upregulated and 2195 downregulated DEGs. DEGs regulate the metabolic and immune pathways in fish, and MS-222 may induce more damage to the fish liver and reproductive organs [21]. The key findings of the current study, which aimed to investigate the MS-222 mechanism in fish anesthesia, are as follows: firstly, MS-222 anesthesia induced a significantly weaker regulatory effect on liver gene networks than on gill tissues at 12 h post-induction. This was evidenced by the fewer DEGs identified in the liver samples (1140; 654 upregulated and 486 downregulated) than in the gill samples (3252; 2309 upregulated and 943 downregulated). In contrast, the number of differential metabolites in gill tissues (173; 74 upregulated and 99 downregulated) was less than that in the liver samples (297; 130 upregulated and 167 downregulated). Secondly, gill tissues prioritized rapid gene expression modulation for anesthetic adaptation, whereas liver tissues relied more on metabolic network reorganization. The transcriptomic analysis of gill tissue revealed significant enrichment in pathways related to (1) cardiac function regulation (cardiac muscle contraction, adrenergic signaling in cardiomyocytes); (2) cellular dynamics (motor protein activity, actin cytoskeleton regulation); (3) metabolic processes (glycolysis/gluconeogenesis, carbon metabolism); and (4) immune responses (toll-like receptor signaling, cytokine–cytokine receptor interaction) and in liver transcriptome alterations primarily involved in (1) protein synthesis mechanisms (endoplasmic reticulum processing, aminoacyl–tRNA biosynthesis); (2) metabolic regulation (PPAR signaling, pyruvate metabolism); and (3) cellular maintenance systems (ribosome biogenesis, spliceosome function).

#### 4.1.2. Gill-Specific Responses

The fish gill is the first target organ for environmental stress, and thus, gill proteomic responses to abiotic stresses have been widely established [30]. In this study, DEGs were enriched in pathways associated with cardiac function regulation (cardiac muscle contraction, adrenergic signaling in cardiomyocytes) and cellular dynamics (motor protein activity, actin cytoskeleton regulation). These observed effects may be associated with MS-222-induced chemical damage to the gill tissue [10,31], as these signaling pathways are typically activated under stress. The activation of these pathways could enhance cardiac function by increasing myocardial contractility and heart rate (via cardiac muscle contraction and adrenergic signaling in cardiomyocytes) [32,33], maintaining cell morphology (actin cytoskeleton regulation) [34], accelerating substance transport, and repairing damaged cellular structures (mediated by motor protein activity) [35], thereby collectively improving the host’s anti-stress capabilities. In addition, the mitogen-activated protein kinase (MAPK) signaling pathway is a critical regulatory pathway in cellular signal transduction, primarily involved in stress response and cell survival, cell proliferation and differentiation, immune and inflammatory responses, and metabolic regulation [36]. In this study, we found that MS-222 may activate the MAPK pathway to trigger stress responses in gill tissues to chemical stimuli, mediate inflammatory reactions to the anesthetic agent in gill immune cells, and synergistically regulate inflammatory responses through the toll-like receptor signaling pathway [37]. Additionally, interactions between the MAPK signaling pathway and the glycolysis/gluconeogenesis metabolic pathway may influence cellular energy status, thereby enhancing the adaptive responses of largemouth bass to the anesthetic agent and maintaining its osmoregulatory homeostasis and immune defense functions [38].

#### 4.1.3. Liver-Specific Responses

The liver is the primary metabolic organ of fish. Transcriptomic analysis of fish liver following MS-222 anesthesia revealed significant alterations in multiple metabolic and immune-related pathways. The most significant enrichment pathways were associated with protein processing in the endoplasmic reticulum, PPAR signaling, metabolism, aminoacyl-tRNA biosynthesis, and pyruvate metabolism. The enrichment of differentially expressed genes involved in protein processing in the endoplasmic reticulum suggests that MS-222 anesthesia may induce endoplasmic reticulum stress, which was similar to the stress induced by lidocaine on PC12 cells [39] and short-term sevoflurane exposure in rats [40]. The liver responds to anesthesia-induced cellular stress by enhancing protein folding and quality control and regulating gene expression related to cytochrome P450 and other detoxification enzyme lines to enhance the metabolism of foreign substances [41]. The PPAR signaling pathway plays a crucial role in regulating lipid and glucose metabolism, as well as inflammation. The alteration in this pathway implies that MS-222 anesthesia might affect metabolic homeostasis in fish, potentially altering lipid storage, glucose utilization, and immune responses [42]. Given the crucial role of metabolic pathways, aminoacyl–tRNA biosynthesis, and pyruvate metabolism in energy production and intermediary metabolism, their enrichment in the identified DEGs indicates that MS-222 anesthesia could influence the energy metabolism of this species.

### 4.2. Metabolomic Remodeling Characteristics

#### 4.2.1. Overall Metabolite Alterations

The difference in metabolites is an important index that reflects the physiological state, environmental adaptability, and health level of fish. The pronounced enrichment of differential metabolites in the gill and liver tissues of largemouth bass following MS-222 anesthesia revealed a multifaceted metabolic reprogramming, reflecting both adaptive and stress-induced responses to chemical exposure. The functional categorization of differential metabolites revealed prominent enrichment in the metabolic pathways of amino and fatty acids. Gill metabolites exhibited predominant involvement in (1) lipid metabolism (fatty acid degradation/elongation, glycerolipid metabolism); (2) aromatic amino acid processing (phenylalanine/tyrosine metabolism); and (3) nucleotide metabolism. Liver metabolites demonstrated stronger associations with (1) essential amino acid pathways (tryptophan, glycine–serine–threonine metabolism); (2) antioxidant systems (ascorbate metabolism); and (3) specialized metabolic processes (caffeine metabolism, alpha-linolenic acid metabolism). In both the gill and liver tissues, 3-aminobenzoic acid ethyl ester methanesulfonate (MS-222), methyl nicotinate, and N-acetyl-L-phenylalanine were significantly upregulated. The detection of 3-aminobenzoic acid ethyl ester methanesulfonate (MS-222) indicates its accumulation in gill and liver tissues. The upregulation of methyl nicotinate may be associated with vasodilation and blood flow alterations induced by MS-222 stimulation, which facilitate the distribution and metabolism of MS-222 [43]. N-acetyl-L-phenylalanine is a phenylalanine metabolite, and its significant upregulation suggests that MS-222 may interfere with phenylalanine metabolism, consistent with the findings of KEGG enrichment analysis, leading to a marked increase in the level of N-acetyl-L-phenylalanine [44]. In the liver, the upregulation of differential metabolites such as Asp-Ser, phenylalanyityrosine, syringetin-3-o-glucoside, and N-lactoyl-phenylalanine indicates that MS-222 may activate the associated metabolic pathways.

#### 4.2.2. Amino Acid-Centric Adaptations

In this experiment, metabolic pathway enrichment analysis revealed that MS-222-induced differential metabolites in the gill and liver of largemouth bass were predominantly associated with the metabolic pathways of several amino acids, including phenylalanine, tyrosine, tryptophan, phenylalanine, glycine, serine, and threonine, as well as tyrosine and tryptophan biosynthesis, which is likely due to their impact on neuronal activity and stress responses. As MS-222 is an anesthetic that inhibits voltage-gated sodium channels, it disrupts neural signaling and induces stress. This activates the hypothalamic–pituitary–interrenal (HPI) axis, leading to elevated cortisol, which modulates amino acid metabolism to meet the need for heightened energy levels and neurotransmitter synthesis [23]. In these metabolic pathways, tyrosine and phenylalanine can be catabolized into intermediates such as fumarate and acetoacetate, which enter the tricarboxylic acid (TCA) cycle to provide energy for coping with metabolic stress during anesthesia [45]. Glycine serves as a precursor for glutathione (GSH) synthesis, and its upregulated metabolic pathway may reflect the organism’s need to scavenge reactive oxygen species (ROS) induced by MS-222 [46]. Threonine is a component of immunoglobulins and mucins, and its enhanced metabolism may suggest mucosal barrier repair or immune responses [47].

#### 4.2.3. Tissue-Specific Energy Strategies

Fatty acid metabolic pathways, such as those involved in fatty acid metabolism, degradation, and elongation, as well as the biosynthesis of fatty acids and unsaturated fatty acids, exhibited significant effects in the gill but not the liver, suggesting that fish may enter the TCA by breaking down fatty acids to produce acetyl-CoA [48], which provides ATP to maintain basal metabolism in gill tissues under MS-222 exposure and starvation. Additionally, our results show that MS-222 exposure activates nucleotide metabolism, enhancing host DNA repair and cell proliferation [49].

## 5. Conclusions

The results of this study revealed that MS-222 anesthesia triggers organ-specific physiological adaptations through the differential regulation of metabolic and immune pathways, which can provide multi-omics insights into the underlying mechanisms of anesthetic responses in fish, highlighting distinct tissue strategies for managing chemical stress.

## Figures and Tables

**Table 1:** Results of transcriptomic sequence assembly.

Sample	Raw Reads	Clean Reads	Clean Base (G)	Error Rate (%)	Q20 (%)	Q30 (%)	GC Content (%)
GCA12_1	47,779,856	45,645,160	6.85	0.03	97.6	93.24	47.33
GCA12_2	48,091,768	46,731,910	7.01	0.03	97.32	92.62	46.16
GCA12_3	46,402,036	45,195,964	6.78	0.03	97.34	92.6	46.76
GMA12_1	46,078,140	43,466,010	6.52	0.03	96.39	91.37	46.54
GMA12_2	46,143,790	44,027,190	6.60	0.03	96.94	92.37	45.40
GMA12_3	47,919,228	46,176,644	6.93	0.03	97.3	92.8	47.41
LCA12_1	54,554,562	53,317,498	8.00	0.03	97.45	92.82	47.88
LCA12_2	54,862,588	53,770,872	8.07	0.03	97.29	92.45	47.59
LCA12_3	50,456,582	49,470,860	7.42	0.03	97.58	93.05	47.43
LMA12_1	51,634,666	50,452,610	7.57	0.03	97.52	92.89	48.88
LMA12_2	50,587,374	49,199,952	7.38	0.03	97.85	93.65	48.64
LMA12_3	52,125,768	50,729,502	7.61	0.03	97.2	92.4	46.22

Note: GCA and GMA indicate gill samples from the control and MS-222 anesthesia groups, respectively; LCA and LMA represent liver samples from the control and MS-222 anesthesia group, respectively. Q indicates the Qphred value.

**Table 2:** Comparison of efficiency statistics.

Sample	Total Reads	Reads Mapped	Unique Mapped	Multi Mapped
GCA12_1	45,645,160	43,950,315 (96.29%)	40,734,042 (89.24%)	3,216,273 (7.05%)
GCA12_2	46,731,910	44,782,783 (95.83%)	41,196,719 (88.16%)	3,586,064 (7.67%)
GCA12_3	45,195,964	43,526,886 (96.31%)	40,055,401 (88.63%)	3,471,485 (7.68%)
GMA12_1	43,466,010	40,121,249 (92.30%)	36,667,547 (84.36%)	3,453,702 (7.95%)
GMA12_2	44,027,190	41,029,375 (93.19%)	38,306,518 (87.01%)	2,722,857 (6.18%)
GMA12_3	46,176,644	44,052,857 (95.40%)	39,506,689 (85.56%)	4,546,168 (9.85%)
LCA12_1	53,317,498	51,649,052 (96.87%)	46,284,750 (86.81%)	5,364,302 (10.06%)
LCA12_2	53,770,872	51,762,201 (96.26%)	45,014,672 (83.72%)	6,747,529 (12.55%)
LCA12_3	49,470,860	47,830,869 (96.68%)	41,344,771 (83.57%)	6,486,098 (13.11%)
LMA12_1	50,452,610	48,712,746 (96.55%)	42,378,751 (84.00%)	6,333,995 (12.55%)
LMA12_2	49,199,952	47,719,632 (96.99%)	43,063,660 (87.53%)	4,655,972 (9.46%)
LMA12_3	50,729,502	48,694,832 (95.99%)	44,096,718 (86.93%)	4,598,114 (9.06%)

Note: GCA and GMA indicate gill samples from the control and MS-222 anesthesia groups, respectively; LCA and LMA represent liver samples from the control and MS-222 anesthesia group, respectively. Q indicates the Qphred value.

## Data Availability

The data presented in this study are available on request from the corresponding author due to privacy reason.

## Supplementary Materials

The following supporting information can be downloaded at: https://www.mdpi.com/article/10.3390/metabo15060349/s1, Table S1: Internal_standard of Metabolome detection used in this study. Table S2: KOG classification of differentially expressed genes of gill between exprimental group and control group; Table S3: KOG classification of differentially expressed genes of liver between exprimental group and control group; Table S4: GO Enrichment of differentially expressed genes of gill between exprimental group and control group; Table S5: GO Enrichment of differentially expressed genes of liverl between exprimental group and control group; Table S6: GO classification of differentially expressed genes of gill between exprimental group and control group; Table S7: GO classification of differentially expressed genes of liver between exprimental group and control group; Table S8: KEGG Enrichment of differentially expressed genes of gill between exprimental group and control group; Table S9: KEGG Enrichment of differentially expressed genes of liver between exprimental group and control group; Table S10: Filtered different metabolites of gill; Table S11: Filtered different metabolites of liver.
